# Emerging Therapeutic Strategies in Asthma: Advances in Treatment, Drug Delivery, Drug Adherence, and Disease Management

**DOI:** 10.1007/s11882-026-01265-6

**Published:** 2026-03-06

**Authors:** Ying Xuan Lim, Yi Ning Choo, Yuet Thong Looi, Yap Wern Chuan, Kai Xin Chiam, Rebecca Shin-Yee Wong, Nancy Choon-Si Ng, Bey Hing Goh

**Affiliations:** 1 Department of Biomedical Sciences, Sir Jeffrey Cheah Sunway Medical School, Faculty of Medical and Life Sciences, Sunway University, Sunway City, Malaysia; 2 Department of Medical Education, Sir Jeffrey Cheah Sunway Medical School, Faculty of Medical and Life Sciences, Sunway University, Sunway City, Malaysia; 3 Faculty of Medicine, University of Cyberjaya, Selangor, Malaysia; 4 Faculty of Education and Liberal Arts, INTI International University, Nilai, Malaysia; 5 Faculty of Health, Australian Research Centre in Complementary and Integrative Medicine, University of Technology Sydney, Ultimo, NSW Australia; 6 Sunway Biofunctional Molecules Discovery Centre, Faculty of Medical and Life Sciences, Sunway University, Bandar Sunway, Subang Jaya, Selangor 47500 Malaysia; 7 Graduate Institute of Cancer Biology and Drug Discovery, College of Medical Science and Technology, Taipei Medical University, Taipei, Taiwan

**Keywords:** Asthma, Biologics, Nanoparticle drug delivery, Gene and regenerative therapies, Digital health technologies

## Abstract

**Purpose of Review:**

This review synthesises evidence published between 2020 and 2025 on emerging therapeutic strategies for asthma, with a focus on three interrelated domains: targeted treatments across asthma endotypes, advances in drug delivery aimed at improving pulmonary deposition and therapeutic efficiency, and technology-enabled tools to support adherence and disease management. We examine how biologics, regenerative and gene-based approaches, nanomedicine platforms, and digital health interventions are reshaping asthma care, while critically appraising their clinical maturity and implementation challenges.

**Recent Findings:**

Advances in precision therapy have substantially improved outcomes for patients with severe asthma, particularly through biologics targeting IgE, IL-5 or its receptor, IL-4/IL-13 signalling, and the upstream epithelial alarmin thymic stromal lymphopoietin. These agents consistently reduce exacerbation frequency and systemic corticosteroid use in selected populations, with favourable short- to medium-term safety profiles. In contrast, regenerative strategies such as mesenchymal stem cell–based therapies, gene-based interventions including miRNA and siRNA modulation, and tolerogenic mRNA vaccines remain largely preclinical, offering mechanistic insight but limited clinical readiness. Nanoparticle-enabled drug delivery systems show potential to enhance pulmonary targeting and controlled release, though evidence is predominantly experimental. In parallel, smart inhalers, digital therapeutics, and environmental monitoring technologies address behavioural and environmental determinants of asthma control by improving adherence, inhaler technique, and trigger identification. However, their real-world impact is constrained by cost, integration into clinical workflows, and limited long-term effectiveness data.

**Summary:**

Asthma management is increasingly moving toward precision, endotype-informed care supported by targeted biologics, advanced delivery systems, and digital self-management tools. While biologics represent the most clinically established advances, regenerative, gene-based, and nanomedicine approaches remain exploratory and require robust long-term evaluation. Key priorities for future research include effective therapies for non–Type 2 and steroid-resistant asthma, improved affordability and equitable access, and integrated care models that combine biomarkers with adherence and environmental monitoring to optimise sustained disease control.

## Introduction

Asthma is a common, chronic inflammatory disorder of the airways affecting individuals across the life course. It is characterised by recurrent wheeze, breathlessness, chest tightness, cough, and mucus hypersecretion, particularly during the night or early morning hours [1]. At a pathological level, asthma reflects a complex and dynamic interplay between immune cells, epithelial signalling pathways, and environmental exposures, resulting in chronic airway inflammation and hyperresponsiveness. Over time, persistent inflammation promotes airway remodelling through processes such as subepithelial fibrosis, smooth muscle hypertrophy, and goblet cell hyperplasia, leading to fixed airflow limitation and reduced responsiveness to standard therapies [2–4].

Despite the availability of effective controller medications for many patients, asthma remains a major global health burden, affecting an estimated 300 million people worldwide and accounting for substantial morbidity, mortality, and healthcare expenditure in severe disease [5]. Importantly, a significant proportion of patients experience persistent symptoms, frequent exacerbations, or corticosteroid-related adverse effects, highlighting fundamental limitations of conventional “one-size-fits-all” treatment paradigms [6]. These shortcomings reflect not only issues of medication adherence and drug delivery efficiency, but also the marked biological heterogeneity of asthma, encompassing distinct inflammatory endotypes with divergent treatment responses [7].

In particular, advances in molecular immunology have clarified the differential success of targeted therapies in Type 2 (Th2-high) asthma, while underscoring the ongoing lack of effective options for Th2-low, neutrophilic, or steroid-resistant disease. This evolving understanding has accelerated a shift toward precision medicine approaches that seek to align therapeutic choice with underlying disease mechanisms, patient behaviour, and environmental context. However, translating these advances into meaningful real-world benefit remains challenging, as pharmacological innovation alone does not address barriers related to drug delivery, long-term adherence, affordability, and integration into routine care.

Against this backdrop, recent breakthroughs in biologic therapies, regenerative and gene-based strategies, nanotechnology-enabled drug delivery systems, and digital health interventions have generated renewed optimism in asthma management. While biologics targeting IgE, IL-5, IL-4 and IL-13, as well as upstream epithelial alarmins, are now clinically established, other emerging approaches such as RNA based therapeutics, stem cell derived interventions, and nanoparticle platforms remain largely preclinical and are associated with significant translational and safety uncertainties. In parallel, smart inhalers, digital therapeutics, and environmental monitoring tools offer novel solutions to longstanding challenges in adherence and trigger avoidance, yet raise questions regarding cost-effectiveness, scalability, and sustained patient engagement.

This narrative review synthesises evidence published between 2020 and 2025 to critically examine emerging therapeutic strategies in asthma across four interconnected domains: targeted pharmacological treatments, advanced drug delivery technologies, adherence-enhancing digital interventions, and integrated disease management tools. Rather than providing a purely descriptive overview, this review evaluates the clinical maturity, mechanistic rationale, and real world applicability of emerging approaches, identifies persistent gaps in asthma care, particularly in non Type 2 disease, and highlights priorities for future research and implementation. A structured narrative literature search was conducted to support this synthesis, as described below.

## Literature Search Strategy

A narrative literature search was conducted to identify recent evidence on emerging therapeutic strategies in asthma. Electronic databases including PubMed/MEDLINE, Scopus, and Web of Science were searched for English-language articles published between January 2020 and March 2025. Search terms included combinations of keywords and Medical Subject Headings related to asthma and emerging interventions, such as “asthma”, “biologic therapy”, “IL-5”, “IL-4”, “TSLP”, “gene therapy”, “RNA-based therapy”, “nanoparticle drug delivery”, “smart inhalers”, “digital therapeutics”, “environmental monitoring”, and “precision medicine”.

Priority was given to clinical trials, real-world observational studies, and high-quality preclinical investigations with clear translational relevance. Additional articles were identified through manual screening of reference lists from relevant reviews and original studies. Studies were selected based on relevance to asthma pathophysiology, therapeutic innovation, and potential clinical or implementation impact. Given the narrative nature of this review, formal quality scoring and meta-analytic techniques were not applied; however, findings were interpreted with consideration of study design, sample size, and translational maturity.

## Cellular and Molecular Pathways of Inflammation

**Fig. 1:**
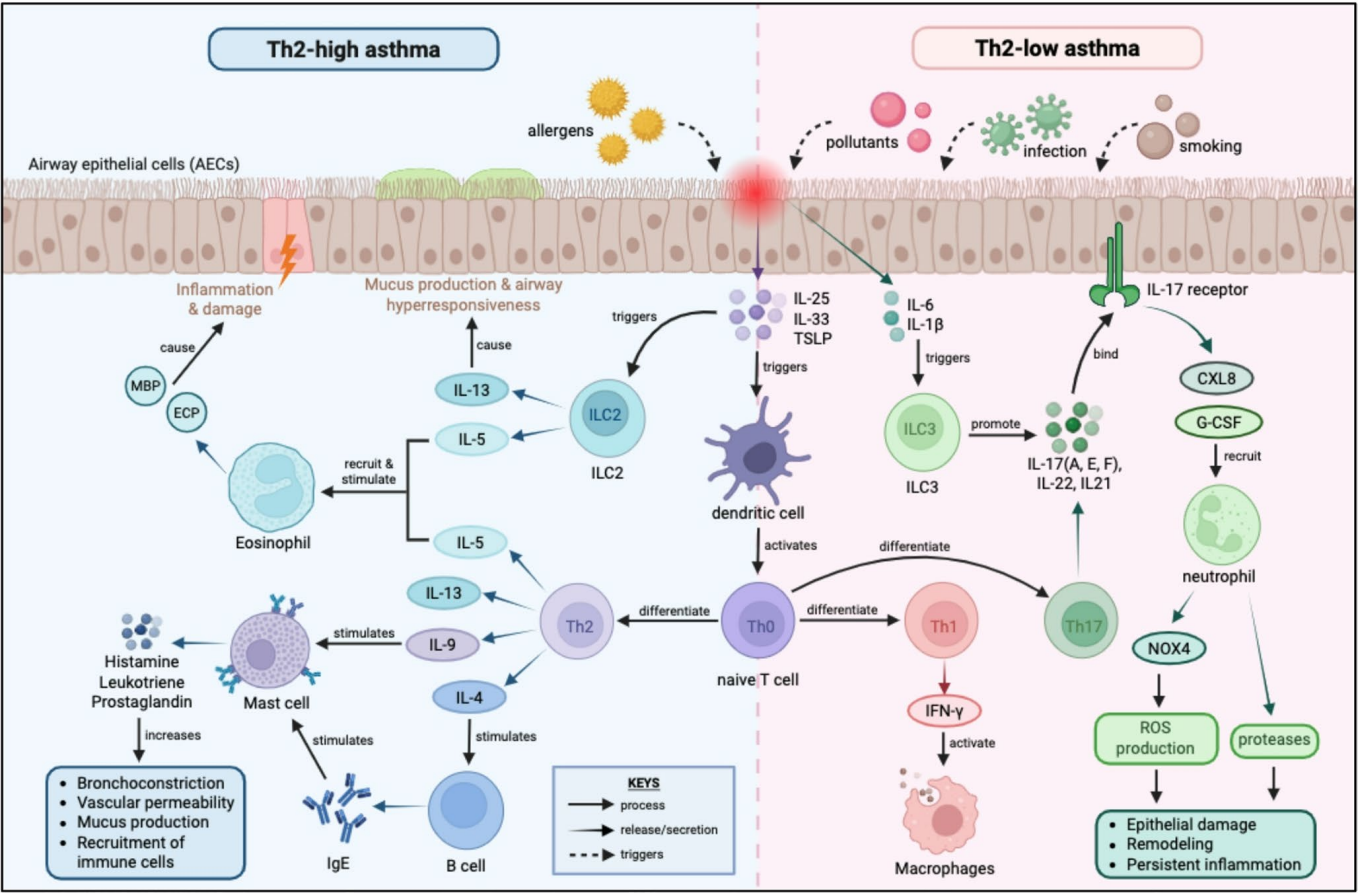
Illustrated pathway involving cellular players and molecular mediators in asthma inflammation, which is separated into Th2-high asthma (blue area) and Th2-low asthma (pink area)

Before examining emerging therapeutic strategies in asthma, it is essential to contextualise them within the underlying cellular and molecular pathways that drive disease heterogeneity and treatment response. Contemporary asthma paradigms recognise at least two dominant inflammatory endotypes, namely Type 2 asthma and non Type 2 asthma, each characterised by distinct immune pathways, clinical phenotypes, and therapeutic vulnerabilities, as illustrated in Fig. 1.

Th2-high asthma represents the most prevalent and therapeutically tractable endotype. It is typically initiated by allergen-induced activation of airway epithelial cells, leading to the release of epithelial “alarmins” such as IL-25, IL-33, and thymic stromal lymphopoietin (TSLP) [8, 9]. These upstream signals promote Th2 cell differentiation via antigen-presenting dendritic cells and directly activate innate lymphoid cells type 2 (ILC2s), bypassing classical antigen presentation pathways [10]. The resulting Th2 dominant cytokine milieu, characterised by IL-4, IL-5, IL-9, and IL-13, drives eosinophilic inflammation, IgE class switching, mast cell activation, mucus hypersecretion, and airway hyperresponsiveness, as summarised in Table 1 [11, 12].

**Table 1:** Summary of Key Cellular Players and Molecular Mediators involved in the Asthma Inflammation Pathway

Cellular players/molecular mediators	Type	Th-endotype involvement	Role in asthma inflammation	Cited Studies
Cellular players				
B cells	Adaptive immune cells	Th-2 high	Produce IgE antibodies under the influence of IL-4	[93]
Dendritic cells in airways	Antigen-presenting cells (APCs)	Both	Capture allergens and present them to naïve T cells to initiate an adaptive immune response	[94]
Eosinophil	Granulocytes	Th-2 high	Release toxic granules (MBP, ECP); cause airway damage and chronic inflammation	[94]
Mast cells	Granulocytes	Th2-high	Release histamine, leukotrienes, and prostaglandins upon IgE activation	[94]
Neutrophil	Granulocytes	Th2-low	Release ROS and proteases; contribute to tissue damage, steroid resistance	[95]
ILC2 (Innate lymphoid cell 2)	Innate immune cell	Th2-high	Amplifies Th2-high inflammation from epithelial cell-released cytokines	[96]
ILC3 (Innate lymphoid cell 3)	Innate immune cell	Th2-low	Secretes neutrophil chemoattractant and drives Th2-low inflammation	[97, 98]
AECs (Airway epithelial cells)	Structural cells	Both	Detects allergens and pollutants, then produces alarmins (TSLP, IL-33, IL-25, IL-6, IL-1β) to activate immune cells	[94]
Th0 (Naïve T cells)	T-helper cells	Both	Undifferentiated T-helper cells until they encounter with specific antigen	[99]
Th1 (T helper 1 cell)	T-helper cells	Th2-low	Drives Th2-low asthma inflammation	[99]
Th17 (T helper 17 cell)	T-helper cells	Th2-low	Drives Th2-low asthma neutrophilic inflammation	[99]
Th2 (T helper 2 cell)	T-helper cells	Th2-high	Drives Th2-high asthma eosinophilic inflammation	[99]
Molecular mediators				
IgE (Immunoglobulin E)	Antibody	Th2-high	Attaches to FcεRI receptors on mast cells and basophils, initiating an intracellular signal cascade to release pro-inflammatory mediators	[100, 101]
IL-25 (Interleukin-25)	Cytokines	Both	Released by triggered AECs, activate the ILC2 and promote cytokine production	[102, 103]
IL-33 (Interleukin-33)	Cytokines	Both	Released by triggered AECs, activate the ILC2 and promote cytokine production	[95]
IL-13 (Interleukin-13)	Cytokines	Th2-high	Induce lipid peroxidation and ferroptosis in airway epithelial cells, which causes mucus production and airway hyperresponsiveness	[104]
IL-4 (Interleukin-4)	Cytokines	Th2-high	Stimulate B cells to produce IgE	[105]
IL-5 (Interleukin-5)	Cytokines	Th2-high	Promotes eosinophil growth and survival	[106]
IL-9 (Interleukin-9)	Cytokines	Th2-high	Stimulates mast cell growth, survival, and the release of inflammatory mediators	[107]
CXL8 (Interleukin-8)	Cytokines	Th2-low	Attracts neutrophil	[108]
IFN-γ (Interferon gamma)	Cytokines	Th2-low	Key Th1 cytokine that activates macrophages	[109]
IL-17(A, E, F) (Interleukin-17 A, E, F)	Cytokines	Th2-low	Promote neutrophilic inflammation	[110]
IL-1β (Interleukin-1β)	Cytokines	Th2-low	Stimulates neutrophilic inflammation and Th17 responses	[95]
IL-21 (Interleukin-21)	Cytokines	Th2-low	Produced by Th17 and modulates inflammation	[111]
IL-22 (Interleukin-22)	Cytokines	Th2-low	Linked with Th17 responses and airway remodelling	[112]
IL-6 (Interleukin-6)	Cytokines	Th2-low	Pro-inflammatory mediator to promote the recruitment and activation of immune cells	[113]
NOX4 (NADPH oxidase 4)	Enzyme	Th2-low	Produce ROS in airway structural cells, contributing to oxidative stress and remodelling	[114]
ECP (Eosinophil cationic protein)	Granule protein	Th2-high	Cytotoxic to airway cells, promoting inflammation and damage.	[115]
MBP (Major basic protein)	Granule protein	Th2-high	Release by eosinophils damages the epithelium and worsens inflammation	[115]
G-CSF (Granulocyte colony-stimulating factor)	Growth factor	Th2-low	Stimulate neutrophil production in bone marrow.	[116]
Histamine, Leukotriene, Prostaglandin	Lipid mediator	Th2-high	Secreted by mast cells to promote bronchoconstriction, mucus secretion, vascular permeability, and inflammation	[11]
TSLP (Thymic stromal lymphopoietin)	Lymphopoietin	Both	Released by pathogen-triggered AECs, and activate the APC pathway to drive the Th2 inflammation.	[9]

Importantly, the relatively linear and hierarchical nature of Th2-high signalling has enabled successful therapeutic targeting. Biologic agents that neutralise IL-5, block IL-4/IL-13 signalling, or inhibit upstream epithelial cytokines have demonstrated consistent reductions in exacerbations and corticosteroid dependence in biomarker-selected populations [13–15]. This endotype therefore exemplifies how mechanistic clarity can translate into precision pharmacotherapy when robust biomarkers and dominant inflammatory drivers are present.

In contrast, Th2-low asthma encompasses a heterogeneous group of patients characterised by non-eosinophilic inflammation, frequently involving neutrophilic or mixed granulocytic patterns. This endotype is commonly associated with environmental irritants, air pollution, smoking, obesity, and recurrent infections [16–18]. Following epithelial activation, cytokines such as IL-6, IL-1β, IL-25, IL-33, and TSLP promote Th1 and Th17 differentiation via dendritic cell signalling. Th1 cells produce interferon-γ (IFN-γ), activating macrophage-driven inflammatory pathways, while Th17 and ILC3 cells secrete IL-17 family cytokines that recruit neutrophils through CXCL8 and granulocyte colony-stimulating factor (G-CSF) [19–21].

Neutrophil-derived proteases and reactive oxygen species—particularly those generated via NOX4—contribute to epithelial injury, airway remodelling, and corticosteroid resistance, hallmarks of Th2-low disease that limit the effectiveness of conventional anti-inflammatory therapies [22]. Unlike Th2-high asthma, this endotype lacks a single dominant cytokine axis, complicating therapeutic targeting and biomarker development. As a result, most Th2-directed biologics show limited efficacy in this population, underscoring a major unmet need in asthma care.

Upstream epithelial targets such as TSLP represent a potential bridge across inflammatory endotypes, as they modulate both Type 2 and non–Type 2 pathways. Tezepelumab has demonstrated efficacy across biomarker-defined subgroups, including patients with low eosinophil counts [23]. However, its effects remain variable, and its long-term impact on airway remodelling and disease modification is not yet established, highlighting the complexity of targeting early immune triggers in heterogeneous disease states.

Together, these mechanistic distinctions provide a biological framework for understanding the differential success of current therapies and motivate the exploration of emerging strategies that extend beyond cytokine blockade. The following sections examine how advances in biologics, drug delivery systems, gene and regenerative therapies, and digital health tools seek to address the limitations imposed by asthma heterogeneity at molecular, physiological, and behavioural levels.

## Emerging Therapeutic Approaches in Treating Asthma

Due to the limitations of conventional asthma therapies, including corticosteroid-related adverse effects, variable treatment responsiveness, and the persistence of steroid-resistant disease in Th2-low asthma, there has been growing interest in alternative therapeutic strategies aimed at improving long-term outcomes. Advances in asthma biology have highlighted that poor disease control often reflects a mismatch between underlying inflammatory mechanisms, drug delivery efficiency, and patient adherence rather than inadequate pharmacological potency alone.

Accordingly, recent research has expanded beyond traditional anti-inflammatory agents to encompass targeted biologics, regenerative and gene-based interventions, advanced drug delivery platforms, and technology-enabled approaches to support adherence and disease management. These strategies differ substantially in their mechanistic rationale, clinical maturity, and translational readiness. In this section, we critically examine emerging therapeutic approaches across these domains, with particular attention to their biological plausibility, current evidence base, and potential limitations. An overview of key developments discussed is summarised in Table 2.

**Table 2:** Overview of Advanced Therapeutic Options for Asthma

Therapeutic Name	Target	Classification	Status	Working principle	References
Omalizumab	IgE	Biologics: Anti-IgE drug	FDA approved	- Treat moderate-to-severe allergic asthma (potentially non-atopic severe asthma) - Reduce asthma exacerbations - Improve lung function - Reduce oral corticosteroid prescription - Drug responsiveness is not associated with pre-treatment biomarkers, but is influenced by genotypes	[24–26, 117, 118]
JYB1904	IgE	Biologics: Anti-IgE drug	Clinical Trial Phase 1a	- Treat allergic asthma - Reduce free IgE	[30]
Mepolizumab	IL-5	Biologics: Anti-IL-5 drug	FDA approved	- Treat severe asthma with eosinophilic phenotype - Reduce asthma exacerbations - Reduce daily corticosteroid - Reduce type 2 inflammation and chemotaxis (IL-4, IL-5, IL-13, eosinophils etc.) - Reduce airway tissue remodeling, sub-basement membrane thickness, airway smooth muscle area and thickness and extent of epithelial damage - Long-term adverse effects include hypersensitivity reactions (anaphylaxis), urticaria, rash and angioedema etc.	[31–34, 119, 120]
Reslizumab	IL-5	Biologics: Anti-IL-5 drug	FDA approved	- Treat severe eosinophilic asthma - Reduce asthma exacerbation rate - Reduce oral corticosteroids	[35, 121]
Benralizumab	IL-5 receptor	Biologics: IL-5 receptor blockade	FDA approved	- Treat severe eosinophilic asthma - Effective in treating late-onset severe asthma (compared to early on-set) - Reduce severe asthma exacerbation rate - Reduce oral/inhaled corticosteroids dosage - Improve airflow and lung hyperinflation - Reduce specialist visit and non-scheduled primary care - Mild adverse effects (influenza-like illness, eczema, rash, nausea, bronchitis) - Rare serious adverse effect: cervical vertebral fracture	[36–39, 122, 123]
Dupilumab	IL-4 receptor	Biologics: Anti-IL-4 receptor blockade	FDA approved	- Treat moderate-to-severe eosinophilic/oral corticosteroid-dependent asthma - Improved lung function - Reduce blood eosinophil - Reduce oral/inhaled corticosteroid - Improve quality of life - Reduce airway mucus hypersecretion - Minimal adverse effects (viral upper respiratory tract infection, bronchitis, pharyngitis etc.)	[40, 41, 43–45, 47, 48, 124–127]
Tezepelumab	TSLP (thymic stromal lymphopoietin)	Biologics: Anti-TSLP drug	FDA approved	- Treat severe, uncontrolled asthma - Reduce asthma exacerbations and symptoms severity - Improved lung function - Reduce type 2 biomarkers - Improve health-related quality of life (reduce overall burden) - Well tolerated with minimal adverse effects (nasopharyngitis, influenza, upper respiratory tract infection, bronchitis etc.)	[49–55, 128–133]
Mesenchymal stem cells (MSCs)	IL-33, IL-25, IL-13, IL-5, IFN-γ/IL-4 ratio	Regenerative therapy	Preclinical study (In vitro and In vivo)	- Reduces lung inflammation - Shifting immune response from Th2-dominant (allergic) toward Th1-favored state	[57]
Bone marrow-derived MSCs (BMSCs)	SDF-1/CXCR4 axis; exosomes	Regenerative therapy	Preclinical study (In vitro and In vivo)	- Boosts BMSCs to migrate to the inflamed lung - Release exosomes to reduce airway inflammation	[61]
Bronchiole-on-a-chip	Simulate asthma by stretching bronchial cells.	Regenerative therapy	Preclinical study (In vitro)	- Showed stronger inflammatory responses like cytokines IL-6 and IL-8, increased compared to healthy controls.	[62]
Induced Pluripotent Stem Cells (iPSCs) and Embryonic Stem Cells (ESCs).	IgE and cytokines (IL-4, IL-5, IL-13)	Regenerative therapy	Preclinical study (In vitro and In vivo)	- Suppressed immune responses - Reduced inflammatory cell infiltration - Decreased levels of IgE and cytokines	[56]
Regulatory T cells (TREG)	Th2 cytokine production	Regenerative therapy	Preclinical study (In vitro and In vivo)	- Preventing T cell proliferation - Reducing the production of inflammatory cytokines	[134]
LNPs-siRNA	TSLP	Gene therapy	Preclinical study (In vitro and In vivo)	- Silence TSLP - Reduce mucus production	[65]
Tf-PEI/Mel-PEI siRNA polyplexes	GATA3	Gene therapy	Preclinical study (In vitro and In vivo)	- Silence GATA3 - Reduce Th2 cytokines and inflammation	[135]
microRNA-182-5p	NOX4	Gene therapy	Preclinical study (In vitro and In vivo)	- Reduce ROS level, mitochondrial damage and epithelial cell apoptosis - Reduce inflammatory response of airway asthma	[63]
Lipid nanoparticles (LNPs)-based mRNA vaccine	Splenic dendritic cell	Gene therapy	Preclinical study (In vitro and In vivo)	- Induce tolerogenic dendritic cells - Suppress inflammation and allergic reactions	[66]
Dexamethasone-neonatal-Fc-receptor-targeted peptide modified polyethylene glycol nanoparticles: FcBP-NP@Dex	-	Nanodrug delivery: PLGA-based nanoparticles	Preclinical (In vitro and In vivo)	- Enhance transepithelial transport of drug - Improve pulmonary retention and distribution of drug - Reduce inflammatory cytokines (TNF-α, IL-13, IL-4)	[67]
Liquorice-glycyrrhizic acid-PLGA	-	Nanodrug delivery: PLGA-based nanoparticles	Preclinical (In vivo)	- Effective drug release (10 h) - Reduce IL-4, IL-5, IL-13, IL-25 - Downregulate Muc5ac mRNA expression - Reduce goblet cell hyperplasia - Reduce mucus hypersecretion - Reduce eosinophilic inflammation	[68]
Berberine- platelet membrane-coated nanoparticle: PM@Ber-NPs	-	Nanodrug delivery: PLGA-based nanoparticles	Preclinical (In vivo)	- Increase IL-12 - Reduce IL-4, IL-5, IL-13), inhibiting lung inflammation	[69]
Baicalein-loaded/encapsulated chitosan nanoparticle	-	Nanodrug delivery: Chitosan-based nanoparticles	Preclinical (In vivo)	- Increase IL-12 - Decrease IL-5 - (Loaded) better reduction on Penh value - (Encapsulated) better reduction of mucus secretion in bronchi	[70]
Zafirlukast-chitosan nanoparticles: ZFR-CS-NPs	-	Nanodrug delivery: Chitosan-based nanoparticles	Preclinical (In vivo)	- Enhance the saturation solubility of the drug - Improve drug release (30 min) - Reduce lung resistance	[71]
Naringenin-glyceryl tristearate-based solid lipid nanoparticles-doxofylline-chitosan-tripolyphosphate-based microspheres: NRG SLN DOX sMS	-	Nanodrug delivery: Solid-liquid nanoparticles	Preclinical (In vitro and in vivo)	- Improve aerodynamic properties of drug (improve drug release: 6 & 12 h) - Reduce serum bicarbonate - Reduce eosinophil counts - Improve respiratory flow rate - Improve tidal volume - Improve bronchial wall lining	[72]
Rhynchophylline-solid lipid nanoparticles: Rhy-SLNs	-	Nanodrug delivery: Solid-liquid nanoparticles	Preclinical (In vivo)	- Improve drug release (6 h) - Reduce ovalbumin-induced airway inflammation - Reduce oxidative stress - Improve airway remodeling - Repress p38 signaling pathway	[73]
Fluticasone propionate and salmeterol xinafoate-L-lysine polyamide nanocarriers: (FP-SAL/Lys-PA) NCs	-	Nanodrug delivery: Polyamide nanoparticles	Preclinical (In vitro)	- Improve pulmonary drug delivery (increase drug release)	[136]
Nano into micro containing rapamycin-nano-polyethylene glycol: NiM_(Rapa/PEG)_	-	Nanodrug delivery: NiM particles	Preclinical (In vitro)	- Protect the drug through the mucus layer until reaching the bronchial epithelium	[137]
Myrtenol-niosome	-	Nanodrug delivery: Noisome-based nanoparticles	Preclinical (In vivo)	- Improve inflammation - Improve oxidative stress - Improve tissue remodeling	[138]
Curcumin and beclomethasone dipropionate-nanosuspensions: CUR + BDP-NS	-	Nanodrug delivery: Multicomponent nanosuspension	Preclinical (In vitro)	- Improve the long-term stability of the drug - Enhance nanocrystals’ apparent solubility of the drug	[139]
Propeller Health	For asthma and COPD patients using existing inhalers	Digital therapeutics (DTx)	FDA-approved	- Attaches a sensor to the inhaler - Tracks usage via Bluetooth - Provides personalized insights via applications	[140, 141]
Teva Digihaler^®^	For asthma and COPD; includes medications like ProAir and AirDuo	Digital therapeutics (DTx)	FDA-approved	- Built-in electronic module records inhalation data - Syncs with mobile app via Bluetooth	[141, 142]
Amiko Respiro^®^	For asthma and COPD; compatible with various inhalers	Digital therapeutics (DTx)	FDA-approved	- Smart sensor tracks inhalation flow and technique - AI-powered app gives feedback and reminders	[142, 143]
Adherium Hailie^®^	For asthma and COPD; supports both reliever and preventer inhalers	Digital therapeutics (DTx)	FDA-approved	- Bluetooth-enabled sensor logs usage and technique - App and web portal support remote monitoring	[142, 144, 145]
Coughy™	Monitors cough frequency in patients with respiratory conditions including asthma	Digital therapeutics (DTx)	-	- Uses smartphone microphone and algorithms to detect and count coughs - Analyses data alongside clinical symptoms like medication use, sleep impact, and activity limitation	[78, 79]
AI Asthma Guard	Designed for asthma management, especially in children and individuals with disabilities	Digital therapeutics (DTx)	-	- Wearable device with physiological and environmental sensors - Uses AI to predict asthma attacks and send an alert through a companion applications.	[78–80]
Personalized alarm systems	Sensors tracked humidity, temperature, particulate matter (PM2.5/PM10), total volatile organic compounds (TVOC), and CO₂.	Environment monitor	-	- Alert system combines lung function data by capturing Peak Expiratory Flow (PEF) via spirometers, and real-time environmental monitoring via custom-built and commercial air-quality sensors to detect asthma risks - System delivers unobtrusive alerts through a smartphone app when potential risks are identified	[87]
Awair Omni indoor air quality monitor	The air quality monitor detected PM2.5 and TVOCs in individual’s homes	Environment monitor	-	- Spirometry was used, and when elevated TVOC or PM2.5 levels were detected by the Awair Omni monitor, it triggered an EMA investigation - The PiLR EMA app sent surveys daily to assess residential air quality and asthma symptoms, with real-time alerts notifying users of poor indoor air quality	[88]
AirBeam sensor	The sensor measures PM2.5, temperature, and humidity	Environment monitor	-	- The sensor measures PM2.5, temperature, and humidity, and provides real-time data via Android AirCasting app. - The AirBeam sensors recorded a wider and more variable range of PM2.5 levels	[89]
Electronic noses	Capture volatile organic compound (VOC) patterns	Environment monitor	-	- Use chemical sensor arrays and pattern recognition to capture volatile organic compound (VOC) patterns in exhaled breath - Identify clinically relevant asthma phenotypes, including eosinophilic and neutrophilic subtypes - Guiding phenotype-specific treatment strategies	[90, 91]

### Biologics Approaches

**Fig. 2:**
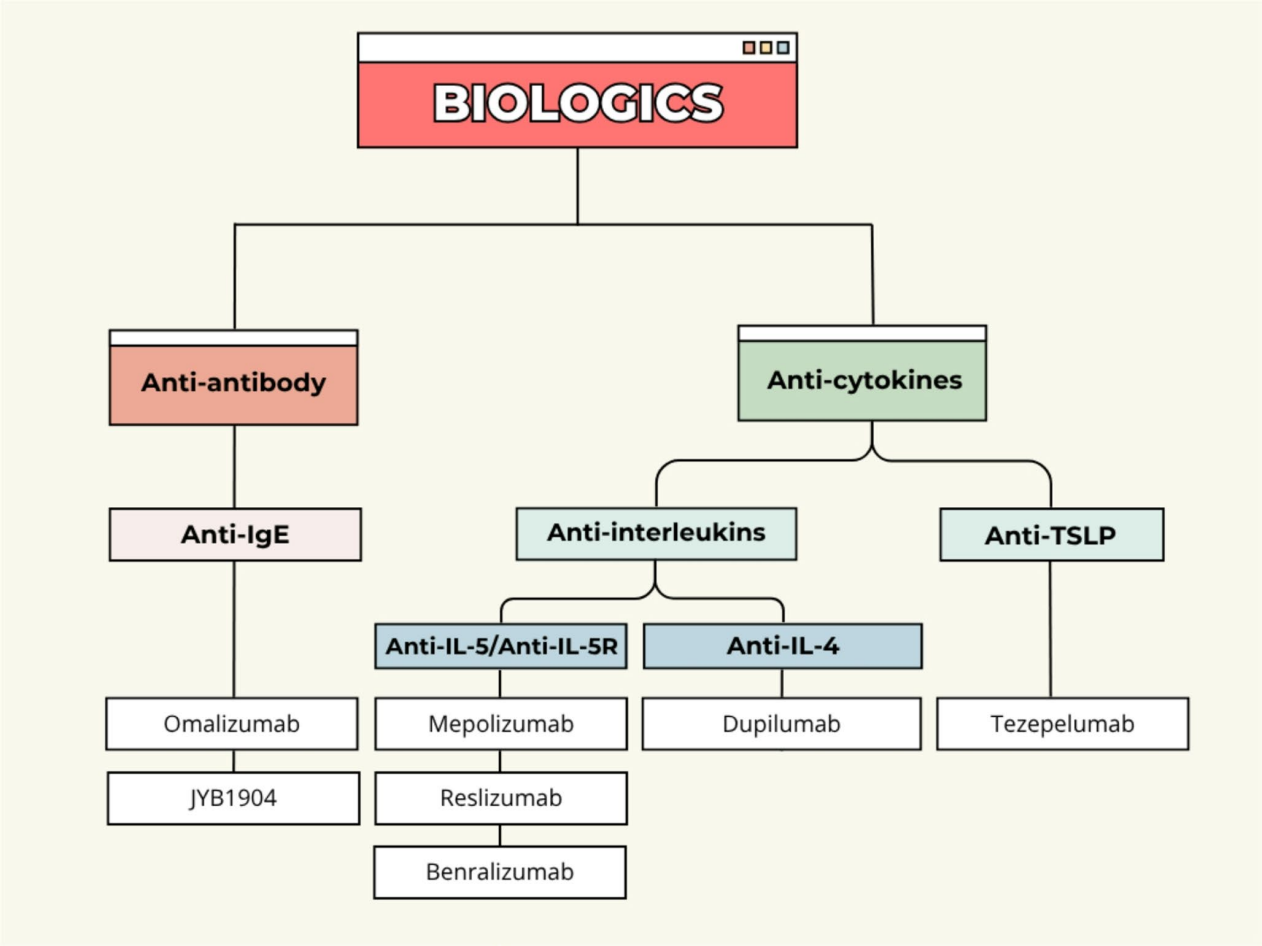
Classifications of biologic approaches used to treat or manage asthma

Biologic therapies represent the most clinically established class of emerging treatments for asthma and have reshaped the management of severe disease by targeting specific immune pathways implicated in Type 2 inflammation. These agents differ in their molecular targets, depth of inflammatory suppression, and applicability across asthma endotypes. Their effectiveness is closely linked to patient selection, biomarker profiles, and disease phenotype, underscoring the importance of precision-based therapeutic strategies. The principal biologic approaches currently used in asthma target IgE, IL-5 or its receptor, IL-4/IL-13 signalling, and the upstream epithelial cytokine TSLP, as summarised in Fig. 2.

#### Anti-IgE Drugs

Omalizumab is a monoclonal antibody targeting circulating IgE and is approved for the treatment of moderate-to-severe allergic asthma [24]. By preventing IgE binding to high-affinity FcεRI receptors on mast cells and basophils, omalizumab attenuates allergen-induced cell activation and downstream inflammatory cascades. Clinical studies and real-world data consistently demonstrate reductions in exacerbation frequency and systemic corticosteroid use, with associated improvements in asthma control [24, 25].

One notable feature of anti-IgE therapy is its relatively broad applicability, as treatment response appears less dependent on baseline demographic characteristics or conventional biomarkers such as blood eosinophil count and total IgE levels [26–28]. This contrasts with cytokine-targeted biologics, which often require stricter biomarker thresholds. Early intervention studies have suggested that omalizumab may modify disease trajectory in young children at high risk of asthma, although long-term prevention and durability of effect remain to be confirmed [29].

Newer anti-IgE agents are being developed to improve pharmacodynamic efficiency and dosing convenience. JYB1904, a recombinant humanised anti-IgE monoclonal antibody, has demonstrated enhanced suppression of free IgE with extended dosing intervals in early-phase clinical studies [30]. While these findings suggest potential advantages over established anti-IgE therapy, their clinical relevance will depend on confirmation of sustained efficacy, safety, and comparative effectiveness in larger and longer-term trials.

#### Anti-IL-5 and Anti-IL-5R Drugs

IL-5 plays a central role in eosinophil maturation, survival, and recruitment, making it a key therapeutic target in eosinophilic asthma. Mepolizumab and reslizumab directly neutralise IL-5, whereas benralizumab targets the IL-5 receptor alpha chain, leading to antibody-dependent cellular cytotoxicity and near-complete depletion of circulating eosinophils. These mechanistic differences may contribute to variability in clinical response and steroid-sparing effects among patients.

Mepolizumab has demonstrated efficacy in patients with severe eosinophilic asthma, with reductions in exacerbation rates, oral corticosteroid requirements, and markers of airway remodelling, including airway smooth muscle thickness and tissue eosinophilia [31–34]. Real-world studies further support its role in improving disease control in patients with persistent eosinophilic inflammation. Reslizumab exhibits broadly similar clinical benefits in appropriately selected populations [35]. Benralizumab has shown particular effectiveness in patients with late-onset severe eosinophilic asthma, with sustained improvements in lung function and substantial reductions in exacerbation frequency and corticosteroid exposure [36–39]. Across anti-IL-5 and anti-IL-5 receptor therapies, safety profiles are generally favourable, with most adverse events being mild to moderate. Rare serious adverse events have been reported, highlighting the importance of continued long-term surveillance in routine clinical practice [40].

#### Anti-IL4 Drugs

Dupilumab is a monoclonal antibody that inhibits IL-4 and IL-13 signalling through blockade of the shared IL-4 receptor alpha subunit, thereby targeting multiple downstream features of Type 2 inflammation [41]. This broader mechanism of action distinguishes dupilumab from biologics that focus primarily on eosinophil suppression. Clinical trials have demonstrated significant reductions in exacerbations and improvements in lung function in children and adults with moderate-to-severe Type 2 asthma, including those with elevated blood eosinophil levels [41, 42].

Beyond inflammatory control, dupilumab has been associated with improvements in mucus hypersecretion, persistent airflow limitation, and structural airway changes, suggesting potential effects on airway remodelling in selected patients [43, 44]. In corticosteroid-dependent asthma, dupilumab enables meaningful dose reduction and, in some cases, discontinuation of systemic corticosteroids while maintaining disease control [45, 46]. The drug is generally well tolerated, with upper respiratory tract infections and injection-site reactions among the most commonly reported adverse effects [47]. Comparative observational data indicate that dupilumab may achieve greater reductions in exacerbation rates and systemic corticosteroid use than anti-IgE therapy in certain populations, although direct head-to-head trials remain limited [48]. Treatment selection should therefore be guided by clinical phenotype, biomarker profile, and comorbid disease rather than efficacy outcomes alone.

#### Anti-TSLP Drugs

Tezepelumab is a human IgG2 monoclonal antibody targeting thymic stromal lymphopoietin, an epithelial-derived cytokine that acts upstream of multiple inflammatory pathways in asthma [49, 50]. By inhibiting TSLP signalling, tezepelumab modulates both Type 2 and non–Type 2 inflammatory cascades, providing broader applicability than cytokine-specific biologics. Clinical trials have demonstrated consistent reductions in exacerbation rates across a wide range of baseline biomarker levels, including patients with low blood eosinophil counts [49]. Treatment with tezepelumab is associated with reductions in Type 2 inflammatory markers such as eosinophils, IL-5, IL-13, and periostin, as well as improved asthma control across seasonal variations [51–53]. These findings support the role of upstream epithelial signalling in asthma pathogenesis. The drug has shown a favourable safety profile in clinical studies, with nasopharyngitis and upper respiratory tract infections being the most commonly reported adverse events [54, 55].

Despite its broad efficacy, it remains unclear whether sustained TSLP inhibition can modify long-term airway remodelling or alter disease progression, particularly in Th2-low asthma. As such, tezepelumab represents an important advance but not a definitive solution for asthma heterogeneity.

### Regenerative Therapies

Regenerative therapies have attracted increasing interest as potential disease-modifying approaches in asthma, particularly in relation to airway remodelling and persistent inflammation that are inadequately addressed by conventional anti-inflammatory treatments. However, current evidence for regenerative strategies in asthma remains largely preclinical, and their clinical applicability has yet to be established.

Mesenchymal stem cells (MSCs) are the most extensively studied regenerative cell type in asthma models, owing to their immunomodulatory properties and capacity to influence tissue repair processes. In murine models of chronic asthma, MSC administration has been associated with reductions in airway inflammation and structural lung damage, particularly when delivered intratracheally [56]. Experimental studies suggest that MSCs can suppress key Type 2 inflammatory mediators, including IL-33, IL-25, IL-13, and IL-5, thereby attenuating eosinophilic inflammation and airway hyperresponsiveness [57–59]. In parallel, MSC treatment has been shown to increase the IFN-γ to IL-4 ratio, indicating a shift away from Th2-dominant immune responses toward a more balanced inflammatory profile.

Despite these promising findings, important limitations remain. The route of MSC administration appears to influence both efficacy and safety, as intravenous delivery has been associated with enhanced fibroblast and myofibroblast migration in injured lung tissue, raising concerns regarding fibrotic remodelling [57]. These observations highlight the complexity of manipulating regenerative pathways in chronically inflamed airways and underscore the need for careful evaluation of delivery strategies.

Bone marrow-derived MSCs (BMSCs) represent a well-characterised MSC subset and have been shown to modulate asthma-related inflammation through homing to injured lung tissue via the stromal cell-derived factor 1 and C-X-C chemokine receptor type 4 signalling axis [60, 61]. Enhanced expression of SDF-1 in inflamed airways facilitates recruitment of CXCR4-expressing BMSCs, while BMSC-derived exosomes further contribute to immunomodulatory effects and reductions in airway remodelling. In preclinical comparisons, BMSCs have demonstrated anti-inflammatory effects comparable to dexamethasone, although the mechanisms and durability of these responses remain uncertain. Modulation of Notch1 signalling has been shown to influence BMSC-induced changes in airway hyperresponsiveness and mucus production, suggesting complex interactions between regenerative pathways and epithelial remodelling processes [61].

In addition to cell-based therapies, advanced in vitro platforms such as lung-on-a-chip models have emerged as enabling technologies for studying asthma pathophysiology and therapeutic responses under human-relevant conditions. These systems replicate key features of the asthmatic airway, including mechanical stretch, bronchoconstriction, and inflammatory signalling, allowing real-time assessment of drug effects. A bronchiole-on-a-chip model incorporating cyclic mechanical strain demonstrated increased production of inflammatory mediators such as IL-6 and IL-8 under asthma-like conditions, while treatment with indacaterol partially reversed these changes and restored smooth muscle cell alignment [62]. Although not a therapeutic intervention itself, such platforms provide valuable tools for preclinical drug screening and for bridging the gap between animal models and clinical trials.

Overall, regenerative approaches in asthma remain exploratory. While preclinical data support their potential to modulate inflammation and airway remodelling, substantial challenges related to delivery, safety, reproducibility, and regulatory translation must be addressed before these strategies can be considered for routine clinical use.

### Gene Therapies

#### siRNA and miRNA Therapy

Gene-based therapeutic strategies aimed at modulating inflammatory signalling have emerged as a potential approach to address molecular drivers of asthma that are not directly targeted by existing pharmacological agents. Among these, microRNA and small interfering RNA platforms have been explored primarily in preclinical models, where they enable selective post-transcriptional regulation of genes implicated in airway inflammation and remodelling.

MicroRNA-182-5p has been investigated as a candidate therapeutic target due to its regulatory effects on nicotinamide adenine dinucleotide phosphate oxidase 4, an enzyme involved in reactive oxygen species generation that is upregulated in asthmatic airway smooth muscle [63, 64]. In experimental asthma models, reduced expression of microRNA-182-5p has been associated with increased oxidative stress, epithelial injury, and inflammatory signalling. Restoration of microRNA-182-5p expression in vitro and in vivo attenuated NOX4 expression, reduced mitochondrial dysfunction and epithelial apoptosis, and suppressed activation of the NOD-like receptor family pyrin domain-containing 3 inflammasome. These effects were accompanied by reductions in Type 2 cytokine production and eosinophilic airway infiltration in animal models.

While these findings highlight the mechanistic potential of microRNA-based modulation in asthma, their translational relevance remains uncertain. Challenges related to targeted delivery, off-target gene regulation, durability of gene silencing, and long-term safety must be addressed before clinical application can be considered.

Small interfering RNA strategies have similarly been explored to silence upstream inflammatory mediators. Inhaled siRNA targeting thymic stromal lymphopoietin has demonstrated efficacy in reducing airway inflammation and mucus production in preclinical models when delivered via ionisable lipid nanoparticles [65]. Cell-specific delivery was achieved through ligand modification enabling binding to intercellular adhesion molecule-1 on airway epithelial cells, facilitating selective uptake and gene silencing. Although this approach offers a targeted means of modulating epithelial-derived inflammatory signalling, it remains confined to experimental settings, and issues related to delivery efficiency, immunogenicity, and repeated dosing have yet to be resolved.

### Tolerogenic mRNA Vaccine

Tolerogenic immunotherapy aims to re-establish immune tolerance to asthma-relevant antigens rather than broadly suppressing inflammatory pathways. Recent advances in mRNA vaccine technology have enabled exploration of this concept in allergic asthma models. A spleen-targeted tolerogenic vaccine incorporating celastrol-enhanced lipid nanoparticles and nucleoside-modified mRNA encoding ovalbumin has been developed to promote tolerogenic dendritic cell differentiation and subsequent induction of allergen-specific regulatory T cells [66]. In murine models of allergic asthma, this approach reduced airway inflammation and disease severity by reshaping the pulmonary immune microenvironment, with minimal cytotoxicity observed in vitro and no overt organ toxicity reported in vivo. These findings support the feasibility of mRNA-based strategies for inducing antigen-specific immune tolerance.

Nevertheless, the clinical applicability of tolerogenic mRNA vaccines in asthma remains highly speculative. The use of ovalbumin as a model antigen limits generalisability to the heterogeneous allergen exposures encountered in human asthma. In addition, long-term immune effects, safety with repeated administration, and scalability of personalised antigen selection represent substantial barriers to translation. As such, tolerogenic mRNA vaccines currently represent a conceptual advance rather than a near-term therapeutic option for asthma management.

## Nanoparticles in Drug Delivery Systems

Nanoparticle-based drug delivery systems have been explored as a strategy to address key limitations of conventional asthma pharmacotherapy, particularly suboptimal pulmonary deposition, rapid mucociliary clearance, and systemic adverse effects. By modifying particle size, surface properties, and release kinetics, nanoparticles can enhance local drug bioavailability within the airways while reducing off-target exposure. To date, most nanoparticle approaches in asthma remain at the preclinical stage, with evidence derived primarily from in vitro studies and animal models.

A range of nanoparticle platforms has been investigated, including poly(lactic-co-glycolic acid) (PLGA)-based nanoparticles, chitosan-based nanoparticles, and solid lipid nanoparticles, alongside more complex systems such as polyamide nanocarriers, nano-into-micro formulations, niosome-based nanoparticles, and multicomponent nanosuspensions, as summarised in Table 2. Across these platforms, proposed advantages include improved drug solubility, controlled release, enhanced penetration of the mucus barrier, and the capacity for co-delivery of multiple therapeutic agents. While these properties are attractive from a pharmacological perspective, their clinical relevance remains to be established.

### PLGA-based Nanoparticles

PLGA-based nanoparticles are among the most extensively studied carriers for pulmonary drug delivery due to their biocompatibility and established use in other therapeutic areas. In asthma models, PLGA nanoparticles have been employed to enhance corticosteroid delivery and prolong pulmonary residence time. For example, dexamethasone encapsulated in neonatal Fc receptor-targeted polyethylene glycol-modified nanoparticles demonstrated improved transepithelial transport and increased lung retention compared with free drug administration [67].

Other PLGA-based formulations incorporating anti-inflammatory compounds such as glycyrrhizic acid and berberine have shown reductions in airway inflammatory markers and eosinophilic infiltration in allergic asthma models [68, 69]. These findings suggest that PLGA nanoparticles can modulate drug pharmacokinetics and local anti-inflammatory effects. However, variability in formulation design, dosing, and outcome measures across studies complicates comparison and limits conclusions regarding optimal carrier characteristics.

### Chitosan-Based Nanoparticles

Chitosan-based nanoparticles have attracted interest for pulmonary delivery due to their mucoadhesive properties and ability to enhance drug solubility. In preclinical asthma studies, chitosan has been used to encapsulate both natural compounds and leukotriene receptor antagonists. Baicalein-loaded and baicalein-encapsulated chitosan nanoparticles demonstrated anti-inflammatory effects in animal models, although differences in release kinetics influenced their relative impact on airway resistance and mucus secretion [70]. Zafirlukast-loaded chitosan nanoparticles have also been evaluated as a potential paediatric asthma formulation, showing improved solubility and rapid drug release with associated reductions in lung resistance in experimental settings [71]. While these findings highlight the versatility of chitosan-based systems, further work is required to assess reproducibility, long-term safety, and suitability for inhaled administration in humans.

### Solid-Liquid Nanoparticles

Solid lipid nanoparticles represent an alternative delivery platform designed to improve drug stability and aerodynamic performance for inhalation. In asthma models, solid lipid nanoparticles have been used to co-deliver bronchodilators and anti-inflammatory agents. A dual delivery system combining naringenin-loaded solid lipid nanoparticles with doxofylline-loaded microspheres demonstrated improved respiratory parameters, including reduced eosinophil counts and enhanced airflow, in preclinical studies [72]. Similarly, rhynchophylline-loaded solid lipid nanoparticles have been shown to attenuate airway inflammation and oxidative stress through suppression of p38 signalling pathways in allergic asthma models [73]. Despite these promising results, solid lipid nanoparticles, like other nanocarrier systems, face challenges related to formulation stability, scalability, and regulatory evaluation.

Collectively, nanoparticle-based drug delivery systems offer a range of theoretical advantages for asthma treatment by enhancing pulmonary targeting and modifying drug release profiles. However, the majority of evidence supporting their use remains preclinical, and significant barriers to translation persist. These include heterogeneity in nanoparticle design, limited long-term safety data, challenges in large-scale manufacturing, and uncertainty regarding regulatory approval pathways. As such, nanotechnology-based delivery platforms should currently be viewed as enabling strategies with potential to complement existing therapies rather than as standalone solutions for asthma management.

## Smart Inhalers and Digital Therapeutics (DTx)

Smart inhalers and digital therapeutics have been developed to address persistent gaps in asthma management that are not adequately resolved by pharmacological advances alone, particularly suboptimal medication adherence and incorrect inhaler technique [74, 75]. These technologies typically incorporate electronic monitoring devices and Internet of Things–enabled sensors into inhaler platforms to capture objective data on medication use, including timing, frequency, and patterns of inhaler actuation and inhalation.

Several commercially available systems are currently used in clinical and real-world settings, including Propeller Health, Teva Digihaler^®^, Amiko Respiro^®^, Adherium Hailie^®^, Coughy™, and AI Asthma Guard [76].

By providing continuous, passive monitoring of inhaler use, these platforms generate granular adherence data that are more reliable than self-reported measures or prescription refill records, thereby offering clinicians a clearer understanding of patient behaviour and treatment gaps [76, 77].

Beyond adherence tracking, some digital inhaler systems integrate feedback mechanisms designed to improve inhaler technique and patient engagement. For example, AI Asthma Guard combines inhalation pattern monitoring with algorithm-driven feedback delivered through connected applications, allowing real-time correction of technique errors and potentially improving pulmonary drug deposition. Coughy™, a smartphone-based application, extends digital monitoring beyond medication use by quantifying cough frequency and exploring its relationship with environmental exposures, symptom burden, and exacerbation risk [78–80]. These approaches reflect a broader shift toward personalised, data-informed asthma management rather than episodic assessment during clinic visits.

Clinical studies have reported meaningful improvements in medication adherence with consistent use of smart inhaler systems, with increases of approximately 30 to 50% observed in selected populations [81–83]. Real-time reminders and feedback appear to play a key role in sustaining adherence and correcting inhaler misuse, which in turn may optimise drug delivery to the lower airways and improve disease control [84]. These benefits highlight the potential of digital therapeutics to complement pharmacological treatment, particularly in patients with poor adherence or frequent exacerbations.

Despite these advantages, several limitations constrain the widespread implementation of smart inhalers and digital therapeutics. Device and subscription costs remain substantial, and limited reimbursement coverage can place financial strain on patients and healthcare systems, particularly in resource-constrained settings [85]. Many existing studies focus on short-term outcomes, leaving uncertainty regarding long-term cost-effectiveness, durability of adherence gains, and impact on disease progression. In addition, integration of digital inhaler data into routine clinical workflows can increase clinician workload and requires supporting infrastructure that is not uniformly available.

Patient acceptance and sustained engagement with digital health tools also vary, with concerns related to usability, data privacy, and technology fatigue reported in some populations [86]. Technical issues such as sensor inaccuracies, false activations, battery dependence, and connectivity limitations may further affect data reliability and user confidence. Collectively, these challenges suggest that while smart inhalers and digital therapeutics offer meaningful opportunities to improve asthma management, their effectiveness at scale will depend on robust technological performance, seamless integration into care pathways, equitable funding models, and stronger evidence demonstrating long-term clinical and economic value across diverse patient groups.

## Digital Environmental Monitors

Digital environmental monitoring technologies have been increasingly explored as adjunctive tools in asthma management, reflecting growing recognition that environmental exposures play a critical role in symptom variability and exacerbation risk. Unlike conventional approaches that rely on population-level exposure estimates, personalised monitoring systems aim to capture real-time, individual-level environmental data and link these exposures to physiological responses and symptoms. Such approaches align with the shift toward personalised asthma care rather than uniform management strategies.

Integrated monitoring systems combining lung function assessment with environmental sensing have demonstrated feasibility in identifying asthma-relevant triggers and supporting early intervention. These platforms typically integrate peak expiratory flow measurements obtained via portable spirometers with continuous monitoring of indoor or ambient environmental parameters, including humidity, temperature, particulate matter, volatile organic compounds, and carbon dioxide levels. When predefined thresholds are exceeded, alerts are delivered through smartphone applications, prompting behavioural modifications or avoidance strategies. Studies evaluating custom-built sensor platforms have reported strong agreement with commercial air quality monitors, with correlations approaching 95% for key environmental parameters, suggesting that low-cost, open-source systems may provide sufficiently accurate data for personalised asthma monitoring [87].

Commercial indoor air quality monitoring systems have also been assessed in real-world asthma management settings. For example, the Awair Omni monitor has been used to detect elevations in particulate matter and volatile organic compounds within residential environments. When integrated with ecological momentary assessment applications and home spirometry, these systems enabled real-time alerts that prompted symptom reporting, lung function testing, and identification of potential exposure sources [88]. Users generally reported high acceptability and perceived educational value, although challenges related to survey timing, device connectivity, and technical reliability were noted. These findings highlight both the promise and the practical constraints of embedding environmental monitoring into daily asthma self-management.

Portable personal exposure sensors offer an alternative approach by capturing high-resolution data on individual exposure to ambient air pollution. Devices such as the AirBeam sensor allow continuous measurement of particulate matter, temperature, and humidity during daily activities, providing insights into spatial and temporal variability that stationary monitoring networks cannot capture. In field studies, these sensors demonstrated acceptable agreement with regulatory monitoring systems while revealing substantially greater variability in personal exposure levels [89]. Although such data can enhance understanding of exposure patterns, translating this information into actionable clinical guidance remains challenging, particularly in the absence of validated exposure thresholds linked to symptom worsening.

In parallel, electronic nose technologies have emerged as a novel, non-invasive approach to asthma phenotyping by analysing volatile organic compound patterns in exhaled breath. These devices employ sensor arrays and pattern recognition algorithms to generate disease-specific breath signatures. Systems such as the Cyranose 320 have demonstrated high diagnostic accuracy in distinguishing asthma from healthy controls and from other respiratory conditions, although their ability to reliably assess disease severity remains limited [90].

Beyond diagnosis, electronic noses show potential for identifying inflammatory phenotypes, including eosinophilic and neutrophilic asthma, and for monitoring changes in airway inflammation over time [90, 91]. Such capabilities could support more targeted treatment selection and monitoring of therapeutic response, particularly in severe or treatment-resistant asthma. However, variability in device performance, limited standardisation of analytical algorithms, and a lack of long-term validation studies currently restrict their clinical application [92]. Collectively, digital environmental monitors and related sensing technologies offer valuable insights into the complex interactions between environmental exposures, physiological responses, and asthma symptoms. While these tools may enhance patient awareness and support personalised management strategies, their effectiveness depends on reliable sensor performance, user engagement, and integration into clinical decision-making frameworks. Further research is required to establish clinically meaningful thresholds, long-term outcomes, and cost-effectiveness before widespread implementation can be recommended.

## Discussion and Future Perspectives

Recent advances in asthma therapeutics reflect a broader shift toward precision medicine, driven by improved understanding of disease heterogeneity and the availability of targeted interventions. The clinical success of biologic therapies illustrates how alignment between inflammatory endotypes, validated biomarkers, and mechanism-specific drugs can substantially improve outcomes in selected patient populations. Blood eosinophil counts, fractional exhaled nitric oxide, and other molecular markers have enabled more rational treatment selection, particularly in Type 2–driven asthma, where biologic therapies have reduced exacerbation rates and corticosteroid dependence.

At the same time, this progress highlights persistent gaps in asthma care. Many emerging strategies remain unevenly effective across disease phenotypes, and patients with non–Type 2 or mixed inflammatory patterns continue to experience limited therapeutic options. While artificial intelligence and data-driven decision support systems offer potential to integrate clinical, biological, and behavioural data, their real-world impact depends on data quality, interpretability, and clinical acceptance. Predictive algorithms may support exacerbation risk stratification and treatment optimisation, but their integration into routine care requires validation across diverse populations and healthcare settings.

Beyond biological efficacy, implementation challenges represent a major barrier to the widespread adoption of emerging therapies. High acquisition costs, particularly for biologics and experimental regenerative or gene-based interventions, limit accessibility and place substantial strain on healthcare systems. Inadequate insurance coverage and reimbursement structures further exacerbate disparities in access, especially in low-resource settings. Moreover, much of the current evidence base is derived from short- to medium-term studies, leaving uncertainty regarding long-term safety, durability of response, and effects on airway remodelling or disease progression. Addressing these gaps will require sustained post-marketing surveillance, pragmatic clinical trials, and policy frameworks that balance innovation with affordability.

Future therapeutic development in asthma is likely to focus increasingly on strategies that extend beyond symptom control toward long-term disease modification. While gene-based and regenerative approaches offer compelling mechanistic promise, current evidence remains largely preclinical, and significant challenges related to delivery, off-target effects, immunogenicity, and regulatory oversight must be resolved. Similarly, advances in nanomedicine may enhance pulmonary drug delivery and targeting, but their translational success will depend on reproducibility, scalability, and demonstrated clinical benefit beyond existing formulations.

In parallel, digital health technologies, including smart inhalers, environmental sensors, and digital therapeutics, provide opportunities to address behavioural and environmental determinants of asthma control that are not amenable to pharmacological intervention alone. When effectively integrated into care pathways, these tools may complement precision pharmacotherapy by improving adherence, identifying triggers, and supporting personalised self-management. However, their long-term value will depend on sustained user engagement, reliable performance, interoperability with clinical systems, and evidence of cost-effectiveness.

In summary, the future of asthma management lies not in a single transformative therapy, but in the coordinated integration of biologically targeted treatments, advanced delivery systems, and data-informed care models. Achieving this will require a balanced approach that prioritises mechanistic insight, clinical relevance, equity of access, and long-term outcomes. Continued interdisciplinary research, coupled with thoughtful implementation strategies, will be essential to translate emerging innovations into meaningful and durable improvements in asthma care.

## Conclusions

Asthma therapeutics have undergone substantial evolution over the past decade, driven by advances in immunology, molecular biology, drug delivery, and digital health. The emergence of biologic therapies has demonstrated that mechanism-based targeting can significantly improve outcomes in carefully selected patients, particularly those with Type 2–driven disease. At the same time, the limited effectiveness of these approaches in Th2-low and corticosteroid-resistant asthma highlights the persistent heterogeneity of the condition and the need for strategies that extend beyond cytokine inhibition alone.

Emerging modalities such as gene-based therapies, regenerative approaches, and nanoparticle-enabled drug delivery offer important mechanistic insights and potential avenues for disease modification. However, the majority of evidence supporting these strategies remains preclinical or confined to early-phase studies. Key challenges related to delivery, long-term safety, reproducibility, and regulatory translation must be addressed before these interventions can be considered viable options for routine clinical practice. As such, their current value lies primarily in advancing understanding of asthma pathobiology and informing future therapeutic design rather than in near-term clinical implementation.

In parallel, digital health technologies, including smart inhalers, digital therapeutics, and environmental monitoring systems, provide practical tools to address non-biological determinants of asthma control such as medication adherence, inhaler technique, and environmental exposure. When thoughtfully integrated into clinical workflows, these technologies have the potential to complement pharmacological advances by supporting personalised, data-informed disease management. Nevertheless, issues related to cost, accessibility, long-term engagement, and health system integration remain significant barriers to widespread adoption.

This review has several limitations. Owing to the rapid pace of innovation, some emerging therapies may be underrepresented or supported by preliminary evidence without long-term clinical validation. In addition, the focus on novel interventions may inadvertently emphasise early positive findings, while established treatments such as inhaled corticosteroids and long-acting bronchodilators remain the foundation of asthma management for the majority of patients. These considerations underscore the importance of interpreting emerging therapies within the broader context of existing, evidence-based care.

Looking forward, meaningful progress in asthma management is likely to depend on the coordinated integration of biologically targeted therapies, advanced delivery systems, digital health tools, and robust long-term clinical evaluation. Addressing unmet needs, particularly in non–Type 2 asthma, will require sustained interdisciplinary collaboration across clinical research, technology development, health policy, and public health. Rather than a single transformative intervention, the future of asthma care will be shaped by incremental advances that collectively enable more predictive, personalised, and participatory management strategies.

## Key References

- Corren J, Menzies-Gow A, Chupp G, Israel E, Korn S, Cook B, et al. Efficacy of Tezepelumab in Severe, Uncontrolled Asthma: Pooled Analysis of the PATHWAY and NAVIGATOR Clinical Trials. *Am J Respir Crit Care Med*. 2023;208:13–24.
  - ○ This pooled analysis strengthens the clinical evidence base for TSLP blockade by demonstrating robust exacerbation reduction across severe, uncontrolled asthma populations. It is particularly important for your review because tezepelumab represents an “upstream” strategy with relevance to both Type 2–high and Type 2–low disease biology.

- Jackson DJ, Heaney LG, Humbert M, Kent BD, Shavit A, Hiljemark L, et al. Reduction of daily maintenance inhaled corticosteroids in patients with severe eosinophilic asthma treated with benralizumab (SHAMAL): a randomised, multicentre, open-label, phase 4 study. The Lancet. 2024;403:271–81.
  - ○ This trial provides high-impact evidence that biologic therapy can enable meaningful inhaled corticosteroid dose reduction while maintaining control in severe eosinophilic asthma. It directly supports your discussion on steroid-sparing, real-world implementability, and the move toward endotype-informed precision management.

- Van De Hei SJ, Kim CH, Honkoop PJ, Sont JK, Schermer TRJ, MacHale E, et al. Long-Term Cost-Effectiveness of Digital Inhaler Adherence Technologies in Difficult-to-Treat Asthma. J Allergy Clin Immunol Pract. 2023;11:3064–3073.e15.
  - ○ This study is important because it addresses a major translational barrier for smart inhalers/digital adherence tools. It supports your “real-world implementation” theme by linking digital monitoring to economic sustainability in difficult-to-treat asthma care pathways.

## Data Availability

Not applicable.
